# The effectiveness of NEPA in the prevention of chemotherapy-induced nausea vomiting among chemo naive patients in an Indian setting

**DOI:** 10.1186/s12885-021-08342-1

**Published:** 2021-05-25

**Authors:** Bharat Vaswani, Palanki Satya Dattatreya, Sagar Bhagat, Saiprasad Patil, Hanmant Barkate

**Affiliations:** 1 Consultant Oncologist and Hematologist, Yashoda Cancer Institute, Secunderabad, India; 2 Consultant Oncologist and Hematologist, Omega Hospital, Hyderabad, India; 3 Medical Services, IF, Glenmark Pharmaceutical limited, B D Sawant Road, Andheri [East], Mumbai, 400099 India

**Keywords:** NEPA, India, Nausea, Vomiting, HEC, MEC

## Abstract

**Background:**

Chemotherapy induced nausea- vomiting (CINV) is considered as the most common, feared and most troublesome side effect of chemotherapy. NEPA (NEtupitant 300 mg + PAlonosetron 0.50 mg) is the first commercially available oral fixed-dose combination (FDC) of two active antiemetic agents in India. The present study was planned to evaluate the effectiveness of NEPA in the real world setting of India.

**Methods:**

This was a multicentric retrospective study conducted in two centers in India. The data of all chemonaive patients, who were prescribed NEPA was analyzed. Effectiveness i.e. complete response and complete protection in controlling overall, acute and delayed phase was analyzed.

**Results:**

A total of 329 patients were enrolled in the study. 260 received highly emetogenic chemotherapy (HEC) regimen and 69 received moderately emetogenic chemotherapy (MEC) regimen. Among all the enrolled patients, complete response in acute, delayed and overall phase was 93, 85.71 and 85.41% respectively; and completed protection was 88.44, 81.76 and 80.54% respectively. Those who received HEC regimen, the completed response and complete protection in overall phase was 84.61 and 79.61% respectively and those who received MEC regimen the completed response and complete control in overall phase was 84.05 and 84.05% respectively.

**Conclusion:**

A single oral dose of NEPA targeting dual pathways showed effective control of nausea-vomiting in patients on the HEC and MEC regimens and had good control over nausea-vomiting in acute, delayed and overall phase of nausea-vomiting.

## Background

In spite of many targeted and biological therapies available in the treatment of various cancers, chemotherapy is still considered the cornerstone in the management algorithm. Unfortunately, side effects due to chemotherapies continue to affect the quality of life of patients. Chemotherapy induced nausea- vomiting (CINV) is one such, which is considered the most common, feared and most troublesome side effect [1]. Decades after discovering the first antiemetic against CINV [2], CINV is still considered the oncologist’s nightmare, as more than 40% patients still experience nausea, vomiting, or both, following the administration of chemotherapy [3]. This may be because of the complex multi-factorial process involved between the receptors and neurotransmitters of the brain and the gastrointestinal tract [4]. With the availability of newer antiemetics, the control over vomiting is substantially good, but nausea, especially in the delayed phase (24–120 h), still continues to be bigger challenge and the unmet need in the present era [5]. The biggest barrier highlighted for the uncontrolled nausea /vomiting is poor adherence to the guidelines, as many studies have suggested that patients receiving chemotherapy do not receive guideline-backed antiemetics [6–8].

NEPA (**NE**tupitant 300 mg + **PA**lonosetron 0.50 mg) is the first commercially available oral fixed-dose combination [FDC] of two active antiemetic agents in India. Netupitant is a new, highly selective NK1RA and palonosetron is a “second-generation” 5-HT3 RA with a longer half-life, as compared to ondansetron, granisetron. The combination has the potential to improve the guideline adherence by targeting two critical pathways involved in the emesis, with a convenient, single oral dose thereby potentially improving treatment compliance, which in turn could improve CINV control. The safety and efficacy of NEPA was evaluated in three pivotal trials, and was found to be well tolerated and safe when used as prophylaxis for acute and delayed CINV [9–11].

Since there is a country specific variation in the response rate to antiemetics prescribed to patients, as highlighted by the multination study (PrACTICE) [6], there was the need to have India specific data, as there was no data on the effectiveness of NEPA in the Indian setting.

Hence the present study was planned to evaluate the effectiveness of NEPA in the real world setting of India.

## Methods

This was a multicentric retrospective study conducted in two centres in India, among 329 patients, after taking approval from the ethics committee. The data of all the chemonaive patients, who were prescribed NEPA prophylactically before first cycle of chemotherapy, and those with a complete data of responses in relation to the control of nausea-vomiting, were analysed in the study. Treatment with Dexamethasone was given as per the treating oncologist preference, which was mostly as 12 mg IV on day 1 (day of chemotherapy) followed by 8 mg BD from day 2 to 4 in patients on HEC and MEC regimen. The responses recorded were the presence or absence of vomiting and nausea. The severity of the nausea was recorded in a visual analogue scale (VAS of 10 mm). Nausea grading < 2.5 mm was considered as no significant nausea (NSN). Data was recorded in a predesigned pro-forma and compiled in Microsoft excel version 2015 and analysed. Descriptive statistics for quantitative variables was represented as mean +/− SD. Qualitative variables (CR and CP) was represented as frequency & percentages.

The following definitions were used for analysing the data:

- Overall complete response (CR-O), defined as no vomiting and no need for rescue medication, at cycle 1 (Time frame: 0–120 h)
- Complete response during acute phase (CR-AP), defined as no vomiting and no need for rescue medication, at cycle 1 (Time frame: 0–24 h) and
- Complete response during delayed phase (CR-DP), defined as no vomiting and no need for rescue medication, at cycle 1 (Time frame: 24–120 h)
- Overall complete protection (CP-O), defined as no significant (< 2.5 mm on VAS) nausea, no vomiting and no use of rescue medication. (Time frame: 0–120 h)
- No nausea - Complete absence of nausea (VAS score 0)

## Result

A total of 329 patients were enrolled in the study. Baseline characteristic showed majority of the patients (*n* = 119) in the age group of 51–60 years, with average age of 53.51 ± 11.98 years. Male to female ratio was 0.85:1. Breast cancer was the most common diagnosis among the enrolled group. Among the 329 enrolled patients, 260 received a highly emetogenic chemotherapy (HEC) regimen and 69 received a moderately emetogenic chemotherapy (MEC) regimen. The commonly prescribed HEC regimen was cisplatin and anthracycline-cyclophosphamide based, and the commonly prescribed MEC regimen was FOLFOX or FOLFIRI. Among all the enrolled patients (*n* = 329), CR-AP, CR-DP and CR-O was 93, 85.71 and 85.41% respectively; whereas completed protection was 88.44, 81.76 and 80.54% in acute, delayed and overall phase respectively. **(**Tables 1 and 2).

**Table 1:** Complete response among enrolled patients

Phases	Complete response (%)		
	Enrolled patients (n = 329)	HEC (n = 260)	MEC (n = 69)
**Acute phase**	93	89.61	98.55
**Delayed phase**	85.71	85.76	84.05
**Overall phase**	85.41	84.61	84.05

**Table 2:** Complete protection among enrolled patients (n = 329)

Phases	Complete protection (%)		
	Enrolled patients (n = 329)	HEC (n = 260)	MEC (n = 69)
**Acute phase**	88.44	85.76	98.55
**Delayed phase**	81.76	81.15	84.05
**Overall phase**	80.54	79.61	84.05

Among those who received HEC regimen (*n* = 260), CR-AP, CR-DP and CR-O was 89.61, 85.76 and 84.61% respectively; whereas completed protection was 85.76, 81.15 and 79.61% in acute, delayed and overall phase respectively. **(**Tables 1 and 2).

Among those who received MEC regimen (*n* = 69), CR-AP, CR-DP and CR-O was 98.55, 84.05 and 84.05% respectively; whereas completed protection was 98.55, 84.05 and 84.05% in acute, delayed and overall phase respectively. **(**Tables 1 and 2).

The incidence of no nausea in the overall phase was 77.5% in enrolled patients, 76.92% in HEC group of patients and 79.71% in MEC group of patients. (Table 3).

**Table 3:** No nausea among enrolled patients (n = 329)

Phases	No nausea (%)		
	Enrolled patients (n = 329)	HEC (n = 260)	MEC (n = 69)
**Acute phase**	86.01	83.46	94.20
**Delayed phase**	78.79	78.07	79.71
**Overall phase**	77.50	76.92	79.71

No significant difference was seen in CR-O (*p* = 0.85), CR-DP (*p* = 0.70) and CP (*p* = 0.70) among patients on HEC and MEC regimen but those on MEC regimen had better (*p* = 0.01) CR-AP as compared to those on HEC regimen. The gender-wise response also showed no difference in the CR-O (*p* = 0.87), CR-AP (*p* = 0.84) and CR-DP (*p* = 0.75) among males and females. The regimen (HEC and MEC) wise comparison also showed no difference in the CR-AP, CR-DP and CR-O among males and females in the study.

## Discussion

NEPA is an oral, single dose fixed dose combination in the management of CINV. The FDC consists of new and high selective NK1 receptor antagonist (NK1RA) (Netupitant) and pharmacologically distinct 5HT3 receptor antagonist (5HT3 RA) (Palonosetron). 5HT3 and NK1 receptor pathways are important in the pathophysiology of CINV, and are responsible for the acute and delayed phases respectively [2, 4]. All the current guidelines, i.e. MASCC/ESMO, ASCO and NCCN recommend the use of 5HT3 RA in the control of the acute phase, and NK1RA in the control of the delayed phase [6–8].

Netupitant is a highly selective and potent NK1 RA with a longer half-life (t1/2 = 96 h) and has higher receptor occupancy as compared to other NK1RA. A positron emission tomography (PET) study conducted to determine the receptor occupancy of netupitant, reported, the RO is long lasting and in the study 300 mg was the lowest dose tested reaching the 90% RO [9, 12]. Palonosetron, a distinct second generation 5HT3 RA, has a unique pharmacological property of receptor internalisation, allosteric binding and the ability to inhibit crosstalk signalling between 5HT3 and NK1 receptor. In comparison to the first generation 5HT3 RA, it has a 30-fold higher affinity and significantly longer half-life [13]. When used in combination, netupitant and palonosetron (NEPA), various in-vitro studies have highlighted synergistic effect in the inhibition of substance P mediated stimulation of NK1 receptor [14] and additive effect on NK1 receptor internalisation [15]. This synergistic action of NEPA was believed to improve the delayed phase of nausea-vomiting which is the current challenge in the management. In addition, the dose of dexamethasone, a CYP3A4 substrate, should be reduced when used along with NEPA as netupitant is a moderate inhibitor of the cytochrome P450 isoenzyme 3A4 (CYP3A4) [16]..

The safety and efficacy of NEPA was evaluated in three pivotal registration trials [1 phase II and 2 phase III] conducted in chemo naïve patients on HEC and MEC regimens. In all the trials, NEPA was administered approximately 60mins prior to chemotherapy. In two efficacy based pivotal trial by Hesketh P [17] and Aapro M [18], the overall complete response (CR) was 89.6 and 74.3% respectively. Thus the result of our study i.e. CR rate of 85.41% is in line with study conducted by Hesketh P [17] and Aapro M [18]. The third pivotal, phase III safety study, conducted by Gralla R [19], reported as secondary endpoint, 81% CR in total population, 84% CR in the subset of patients on HEC regimen and 80% in patients on MEC regimen. Our study is in line with the result of above study, where the CR in patients on HEC and MEC regimen was 84.61 and 84.05% respectively. Another registration trial conducted in Chinese patients by Zhang L [20], too, reported CR in the same range, i.e. 73.8% in the overall phase and 84.5 and 77.9% in the acute and delayed phase. Thus the overall CR in the above studies ranges from 73 to 90%.

The commonly prescribed HEC regimen in our study was cisplatin and anthracycline-cyclophosphamide based, and the commonly prescribed MEC regimen was FOLFOX or FOLFIRI. The treatment with NEPA thus had overall good control in both the HEC and MEC regimens, because it follows the guideline [6–8] recommendation of using 5HT3 RA for acute phase and NK1RA for delayed phase in patients on the HEC and MEC regimens with associated risk factors.

Despite much advancement in the recent years, nausea is still considered the most bothersome symptom, many patients grade nausea over vomiting as the worst side effect of chemotherapy and delayed nausea to be more troublesome than acute nausea [21]. The biggest problem highlighted with nausea is its subjective nature and hence difficult to define and control. Also, many of the clinical trials have not considered nausea in the primary or secondary endpoint. Our study recorded nausea on a VAS of 100 mm and the incidence of no significant nausea (< 25 mm) was used for further analysis to determine complete protection. The overall CP in our study was 80.54% with better CP in patients on MEC (84.05%) than in the HEC (79.61%) regimen. Complete absence of nausea was reported in 77.5% enrolled patients in our study. The dose ranging study by Hesketh P [17] reported that with 300 mg netupitant, the overall CP rate was 83%, which was similar to our study, whereas CP in our study was much better than the study conducted by Aapro M [18] in AC regimen patients, where CP reported was 63.8% in enrolled patients. The above results with our study and two pivotal studies indicate that NEPA had good control of nausea. This can be because of the synergistic action of palonosetron and netupitant in the prevention of cross talk phenomenon, additive effective of receptor internalisation and higher and longer receptor occupancy of netupitant.

In India, the current management includes the use of aprepitant or fosaprepitant as a NK1 RA. Fosaprepitant, as a single IV dose of 150 mg on day 1 or a 3-day dosing regimen of oral aprepitant (125 mg on day 1, 80 mg on days 2 and 3). A phase III RCT conducted by Maru A [22] on Indian subset of population on cisplatin based regimen reported CR-O of 77.1 and 73.4% with fosaprepitant and aprepitant arms respectively, with CR-AP as 94.2 and 90.1% and CR-DP as 77.7 and 73.9% in fosaprepitant and aprepitant arms respectively. Though a double blinded RCT comparing NEPA with Aprepitant/ Fosaprepitant will highlight the superiority of the regimen, the initial report with this indirect comparison suggests NEPA as a better option to the available NK1RA, especially with its effectiveness in managing the delayed phase of nausea vomiting and compliance associated with its use.

The importance of having an effective and safe control of nausea-vomiting in chemonaive patients on the first cycle of chemotherapy was highlighted in study conducted by Molassiotis A [23], where poor control in the first cycle was associated with almost 6.5 times the risk of CINV in the second cycle and almost 14 times in the third cycle. This is because of the anticipatory effect associated to poor control in the previous cycle, which becomes difficult to manage.

## Conclusion

The single oral dose of NEPA targeting dual pathways showed effective control of nausea-vomiting in patients on the HEC and MEC regimens. Its synergistic effect had good control over nausea in both the delayed and the overall phase. The single oral will help in improving compliance with the already complex antiemetic regimen and thus with the anti-cancer therapy. This can also help in improving the guideline adherence in the therapy.

## Data Availability

The datasets used and/or analysed during the current study available from the corresponding author on reasonable request.

## Abbreviations

CINV: Chemotherapy-Induced Nausea Vomiting
NEPA: Netupitant 300 mg + Palonosetron 0.50 mg
HEC: Highly Emetogenic Chemotherapy
MEC: Moderately Emetogenic Chemotherapy
FDC: Fixed-dose combination
NK1RA: Neurokinin-1 Receptor antagonist
5HT3RA: 5-hydroxytryptamine 3 receptor antagonist also known as serotonin receptor antagonists
CR-AP: Complete response in Acute Phase
CR-DP: Complete response in Delayed Phase
CR-O: Complete response in Overall Phase
CP-AP: Complete Protection in Acute Phase
CP-DP: Complete Protection in Delayed Phase
CP-O: Complete Protection in Overall Phase
RCT: Randomised Controlled Trial
VAS: Visual Analogue Scale
NSN: No Significant Nausea
